# Supplementary material for: Imbibition-induced selective wetting of liquid metal
Source: Nat Commun. 2022 Aug 13;13:4763. doi: 10.1038/s41467-022-32259-3 (PMC9376080; doi:10.1038/s41467-022-32259-3)
Supplement: Supplementary file 1 — Supplementary Information [file 41467_2022_32259_MOESM1_ESM.pdf]

Supplementary Information

Imbibition-Induced Selective Wetting of Liquid Metal

Ji-Hye Kim[1,7], Sooyoung Kim[2,7], Hyeonjin Kim[3], Sanghyuk Wooh[3], Jiung Cho[4], Michael D. Dickey[5], Ju-Hee So[6]*, Hyung-Jun Koo[2]*

[1] Department of Energy and Chemical Engineering, Seoul National University of Science & Technology, Seoul, 232 Gongneung-ro, Nowon-gu, Seoul 01811, Republic of Korea

[2] Department of Chemical & Biomolecular Engineering, Seoul National University of Science & Technology, 232 Gongneung-ro, Nowon-gu, Seoul 01811, Republic of Korea

[3] School of Chemical Engineering & Materials Science, Chung-Ang University, 84 Heukseok-ro, Dongjak-gu, Seoul, 06974, Republic of Korea

[4] Western Seoul Center, Korea Basic Science Institute, 150 Bugahyeon-ro, Seoul, 03759 Republic of Kore

[5] Department of Chemical and Biomolecular Engineering, North Carolina State University, Raleigh, NC, 27695 USA

[6] Material & Component Convergence R&D Department, Korea Institute of Industrial Technology, Ansan-si, 15588, Republic of Korea

[7] These authors contributed equally: Ji-Hye Kim, Sooyoung Kim.

Correspondence and requests for materials should be addressed to J.-H. So (jso@kitech.re.kr) or to H.-J. Koo (hjkoo@seoultech.ac.kr).

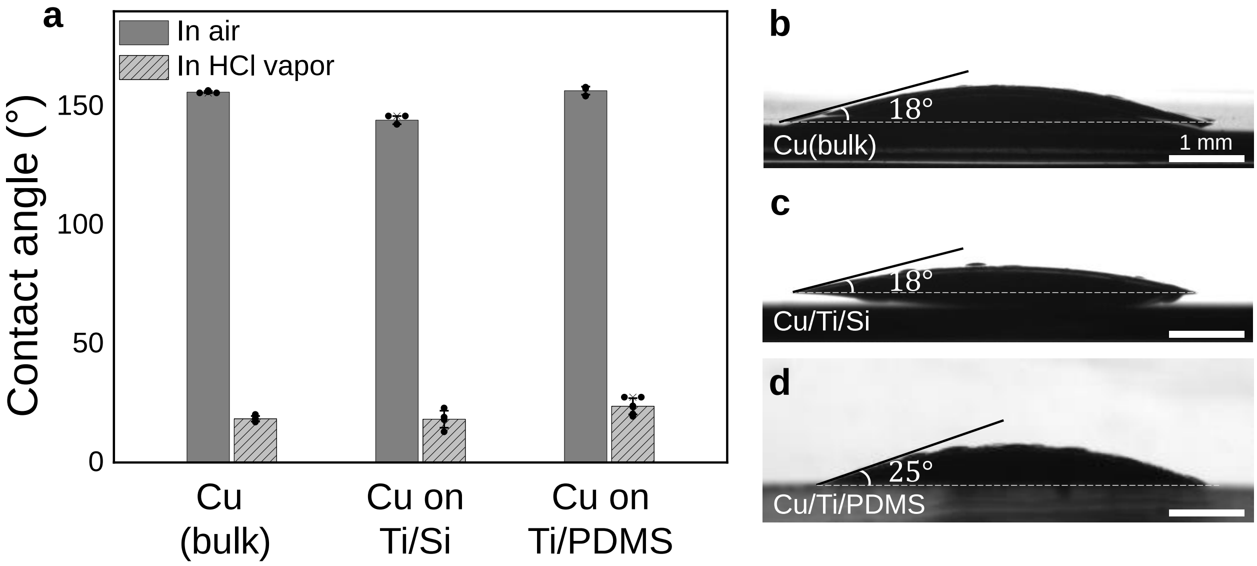

Supplementary Figure 1. Contact angles of EGaIn on copper surfaces. a Contact angles of EGaIn in air and HCl vapor measured on the bulk copper plate and the deposited copper metal films. **b–d** Side-view images of EGaIn in HCl vapor placed on the copper surfaces prepared in different ways: **b** Bulk copper plate; **c** Copper thin film deposited on Si; **d** Copper thin film deposited on PDMS. The thickness of the deposited copper film is 100 nm. A 10-nm thick titanium film was deposited as the adhesion layer. Data are presented as mean ± standard deviation ($n > 3$).

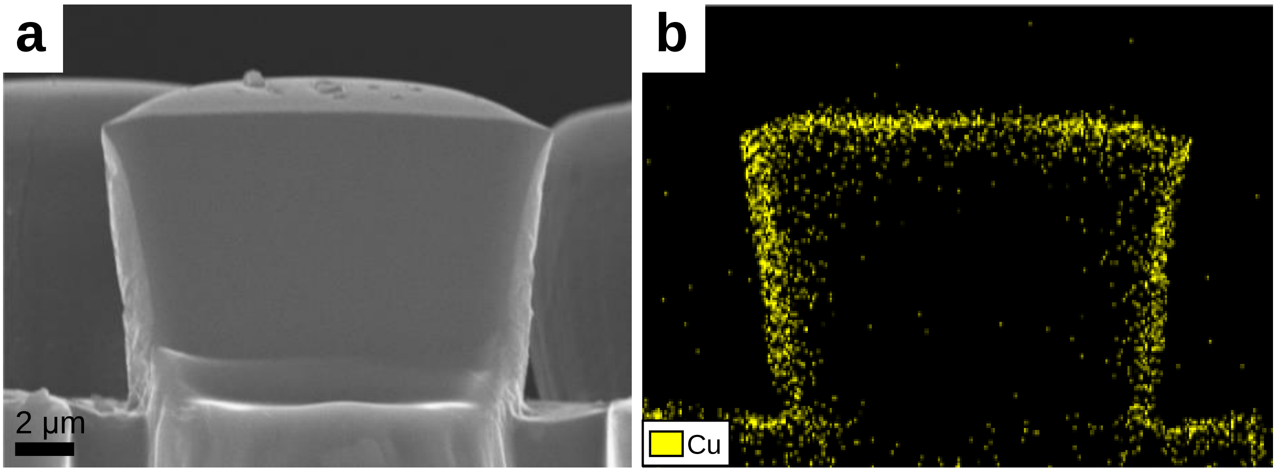

Supplementary Figure 2. Conformality of the copper deposition. a Side-view scanning electron microscope (SEM) image of the copper-sputtered PDMS posts. **b** Spatial distribution of copper in **a** by energy dispersive X-ray spectroscopy (EDS) analysis.

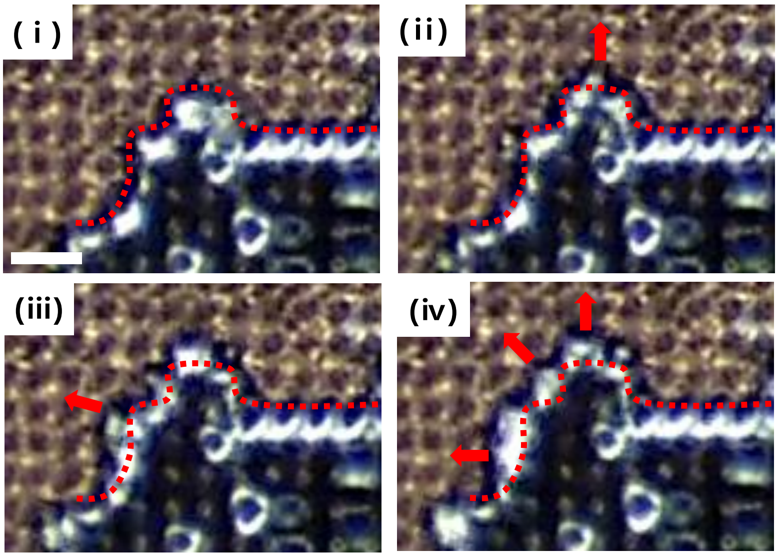

Supplementary Figure 3. Snapshots of imbibition wetting of EGaIn on the microstructured metallic surface after HCl treatment. The images were taken from Supplementary Movie 2. The red dotted line indicates the initial boundary in (i) and the red arrows show the propagation direction of imbibition-induced wetting of EGaIn. Scale bar is 100 µm.

Supplementary Discussion 1. Derivation of θ_c for imbibition

Imbibition is affected by surface morphology and has been established by Bico et al[1]. According to Bico et al., interfacial energy (d*F*) per unit length (d*x*) is defined by the following equation:

$$\mathrm{d}F = (\gamma_{SL} - \gamma_{SV})(r - \phi_s)\mathrm{d}x + \gamma(1 - \phi_s)\mathrm{d}x,$$

where γ_{SL} denotes the surface energy between solid and liquid, γ_{SV} that between solid and vapor, and γ that between liquid and vapor; r represents roughness, and ϕ_s $(= \pi R^2/d^2, R$ denotes the radius of the post and d the lattice distance) is the fractional area of top of the posts. According to the Young's relation, γ_{SV} is the same as $\gamma_{SL} + \gamma\cos\theta_0$, where θ_0 denotes the contact angle on a flat surface. Imbibition could occur when $\mathrm{d}F$ is negative, which fulfills the following condition:

$$\cos\theta_0 > \frac{1 - \phi_s}{r - \phi_s}$$

Thus, we can define the critical contact angle, θ_c:

$$\cos\theta_c = \frac{1 - \phi_s}{r - \phi_s}$$

Supplementary Discussion 2. Derivation of $\theta_{c,pin}$ for imbibition

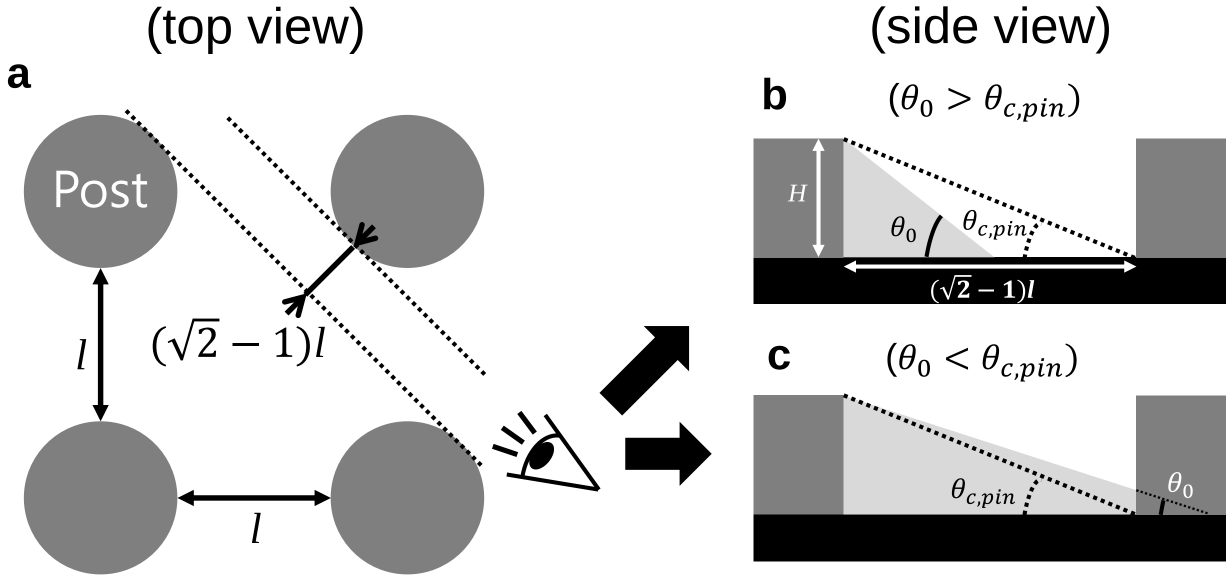

Supplementary Figure 4. Schematics for calculation of $\theta_{c,pin}$. a A comparison of the distance between the diagonal and axes (top view). The diameter of posts and spacing between posts are the same as *l*. **b, c** Side view of the diagonal: **b** In the case of $\theta_0 > \theta_{c,pin}$, where the liquid is pinned before the next post. **c** In the case of $\theta_0 < \theta_{c,pin}$, where the liquid arrives at the next post.

The mechanism of how the liquid can advance through the patterned surface has been explained by Courbin et al[2]. According to Courbin et al., imbibition can occur more easily and quickly through the diagonal than the axes. This is because the distance between the posts toward the diagonal, which is $(\sqrt{2}-1)l$, is shorter than that of the axes, which is l (Supplementary Figure 2a). Thus, regarding the critical contact angle for pinning,

$$\theta_{c,pin} = \arctan\left(\frac{H}{(\sqrt{2}-1)l}\right).$$

We can consider two cases via the diagonal direction. One is that liquid cannot wet the next post and gets pinned on the substrate. Supplementary Figure 2b shows that liquid cannot reach the next post because θ_0 is larger than $\theta_{c,pin}$. The other is that liquid can wet the next post sufficiently and advance. Supplementary Figure 2c indicates that liquid arrives at the next post because θ_0 is smaller than $\theta_{c,pin}$. Therefore, pinning is expected to disappear if θ_0 decreases for the liquid or the aspect ratio $\left(\frac{H}{(\sqrt{2}-1)l}\right)$ increases.

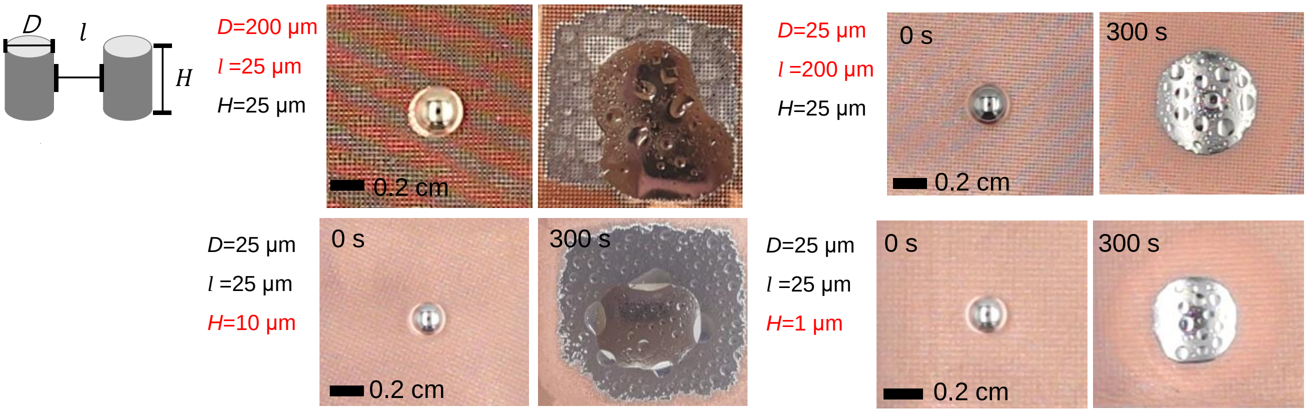

Supplementary Figure 5. Imbibition-induced wetting behavior of EGaIn depending on the dimensions of posts.

Supplementary Table 1. θ_c, $\theta_{c,pin}$, L_c and L as a function of the dimensions of posts.

D (μm)	l (μm)	H (μm)	θ_0	θ_c	$\theta_{c,pin}$	L_c	L (l/H)	Imbibition
200	25	25	~25°	57°	68°	5.2	1	○
25	25	10		44°	44°		2.5	○
25	200	25		16°	17°		8	×
25	25	1		16°	5°		25	×

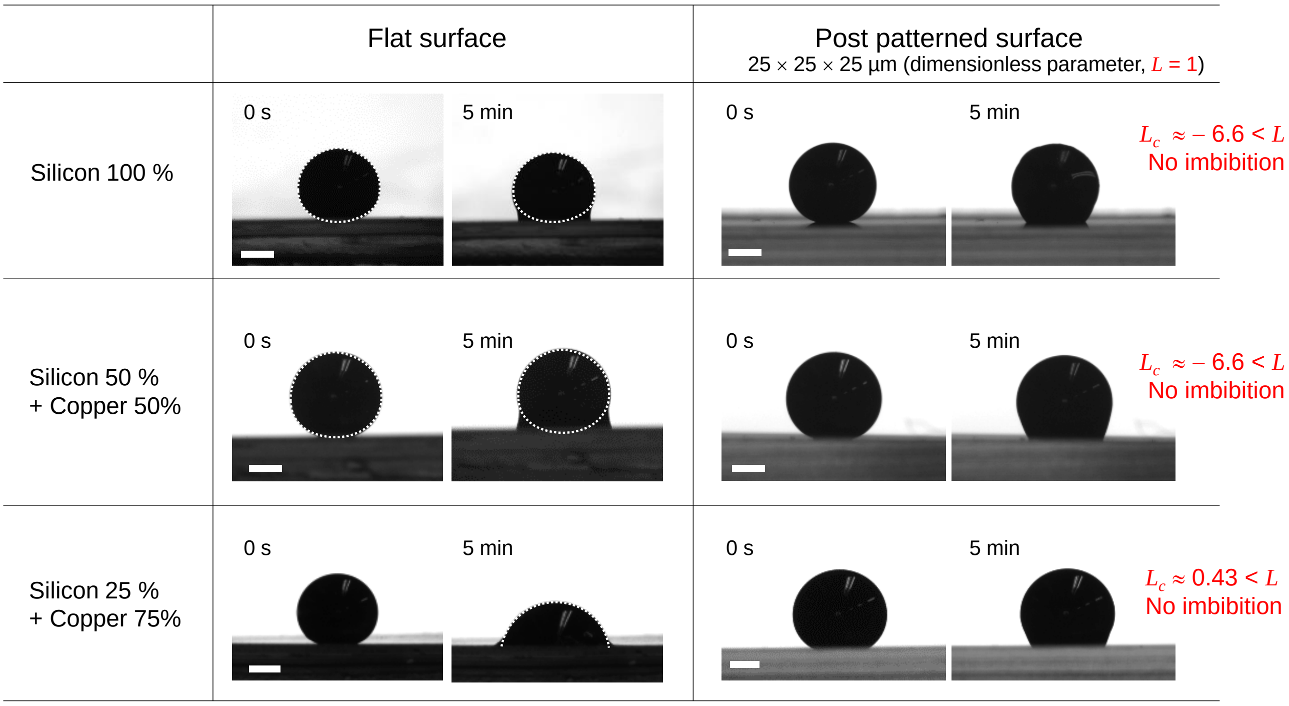

Supplementary Figure 6. Wetting properties of EGaIn on Cu/Si binary surfaces without and with post patterns ($D = l = H$ = 25 µm, L = 1) in the presence of HCl vapor. Scale bars are 1 mm.

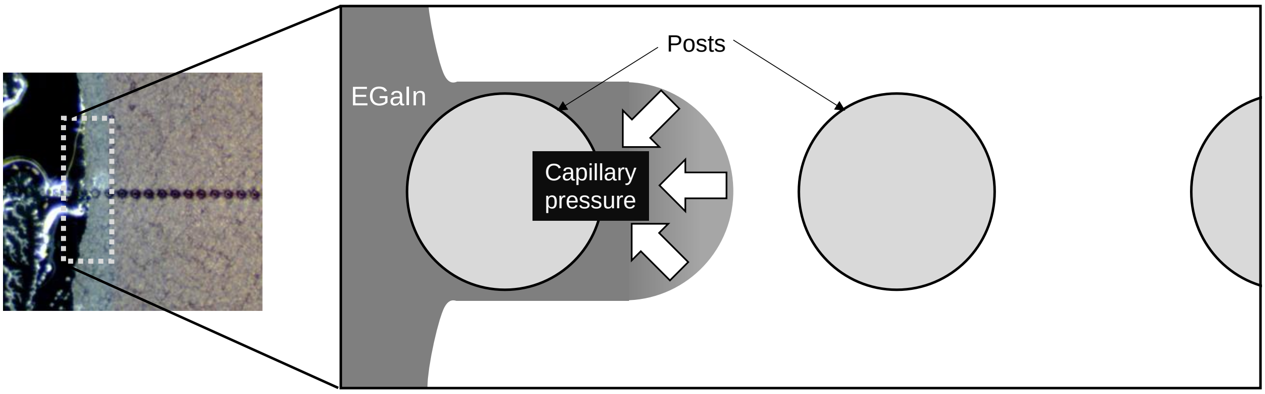

Supplementary Figure 7. Schematic of the capillary pressure at the front of EGaIn on the one row of posts.

Supplementary Table 2. Comparison of studies on coating and patterning of GaLMs on metallic substrate.

Liquid metal	Substrate	Deposition method of liquid metal	External force	Minimum contact angle [1)]	Required time for minimum contact angle	Selective wetting	Reference
Ga	Au/PDMS	Thermal evaporation	Required (vacuum and thermal energy)	0°	– [2)]	×	3
Ga	Au/PDMS (micro-structured)	Thermal evaporation	Required (vacuum and thermal energy)	0°	– [2)]	×	4
Galinstan	Cu/SEBS	Direct contact (rolling)	Required (mechanical energy)	0°	– [2)]	○ by metal-metal interaction [3)]	5
Galinstan	Au/PDMS	Direct contact (rolling)	Required (mechanical energy)	0°	– [2)]	○ by metal-metal interaction [3)]	6
EGaIn	Porous Cu foam	Electrochemically enabled reactive wetting	Required (electrical energy)	Probably 0°	– [2)]	×	7
EGaIn<br>Galinstan	In foil<br>Sn foil	Spontaneous wetting	Not required	10°<br>20°	8.2 s<br>4 days	×	8
Galinstan	Cu sheet treated with $CuCl_2$	Spontaneous wetting	Not required	4.3°	200 s	○ by metal-metal interaction [3)]	9
**EGaIn**	**Cu/PDMS (micro-structured)**	**Spontaneous wetting enhanced by imbibition**	**Not required**	**0°**	**~ 40 s**	○ **directed by microstructure**	**This work**

[1)] 0° means a "complete wetting" was achieved.

[2)] Not applicable. The required time for these works strongly depends on the deposition process condition. The required times in this table have been considered only for the cases of spontaneous wetting.

[3)] The selective wetting occurs along the pre-deposited patterns of metal.

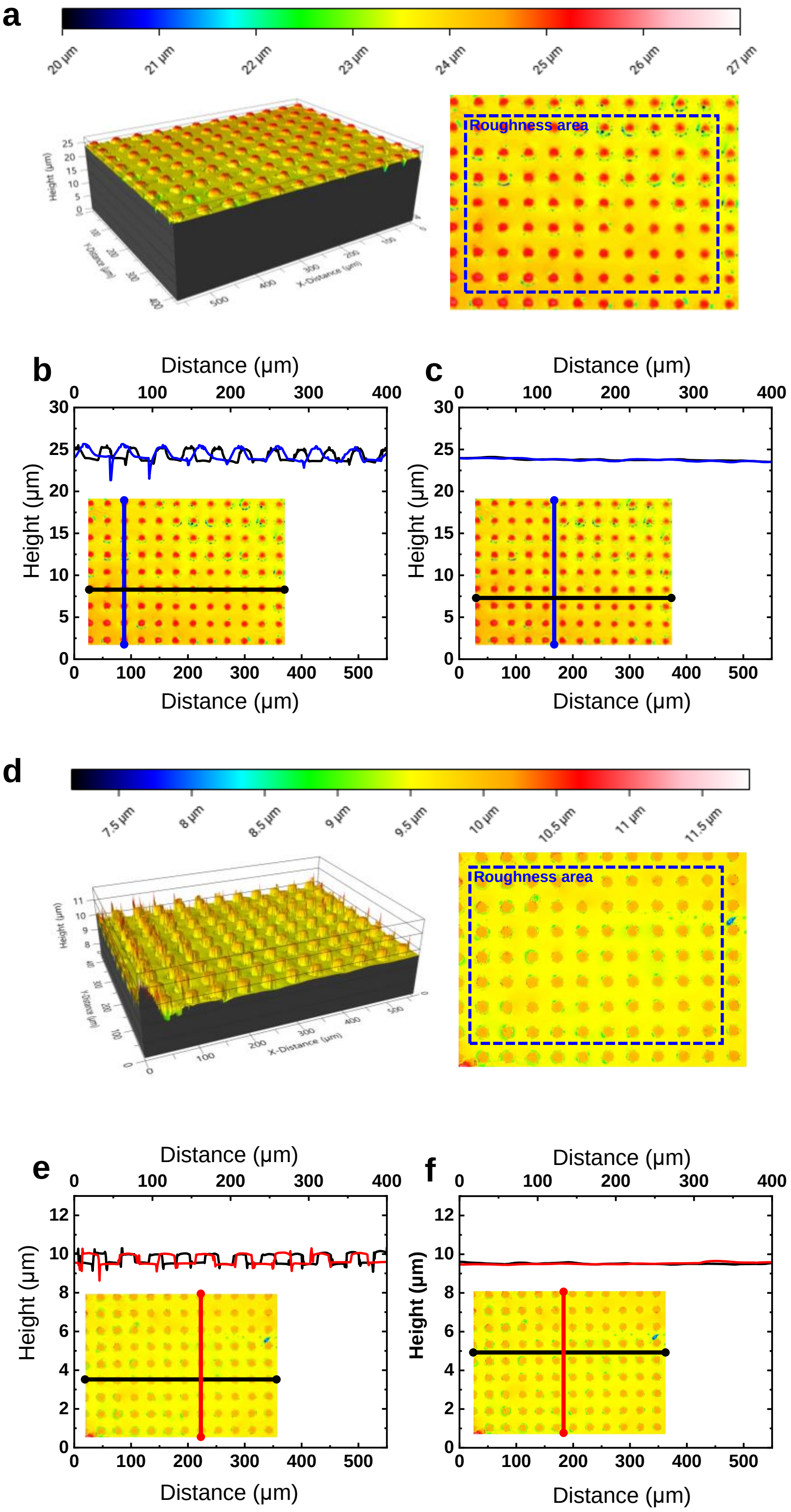

Supplementary Figure 8. Surface topographies of EGaIn/Cu/PDMS with different height of posts. a 3D and 2D surface topography and of EGaIn/Cu/PDMS with posts of 25 µm height. **b, c** Surface profiles of **a**. **d** 3D and 2D surface topography and of EGaIn/Cu/PDMS with posts of 10 µm height. **e, f** Surface profiles of **d.**

Supplementary Table 3. Surface roughness of EGaIn/Cu/PDMS in Supplementary Figure 8.

Height of posts	S_a (μm)	S_q (μm)
10 μm	0.1418	0.2041
25 μm	0.3235	0.4833

* S_a: Arithmetic surface roughness; S_q: Root mean square surface roughness

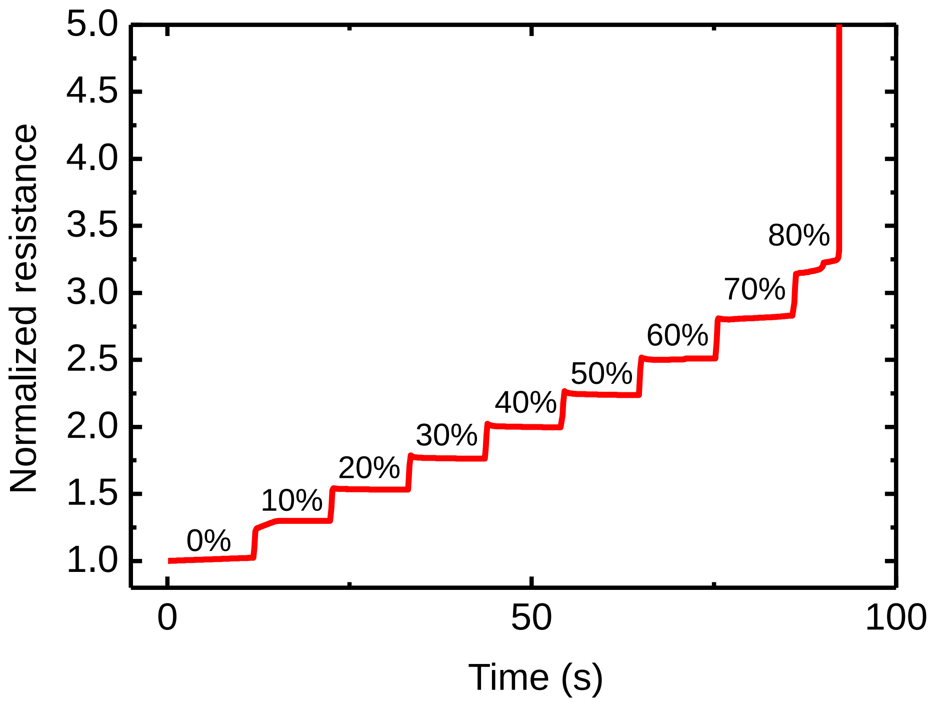

Supplementary Figure 9. The normalized resistance change of EGaIn/Cu/PDMS depending on strain in the range of 0% to 80%. With more than 80% strain, the PDMS was broken and resistance measurement was not possible.

Supplementary References

1. Bico, J., Thiele, U. & Quéré, D. Wetting of textured surfaces. *Colloids Surf., A* **206**, 41-46 (2002).
2. Courbin, L., et al. Imbibition by polygonal spreading on microdecorated surfaces. *Nat. Mater*. **6**, 661-664 (2007).
3. Hirsch, A., Michaud, H. O., Gerratt, A. P., De Mulatier, S. & Lacour, S. P. Intrinsically stretchable biphasic (solid-liquid) thin metal film. *Advanced materials* **28**, 4507-4512 (2016).
4. Hirsch, A. & Lacour, S. P. A Method to form smooth films of liquid metal supported by elastomeric substrate. *Advanced science* **5**, 1800256 (2018).
5. Zhu, Z., et al. Fully solution processed liquid metal features as highly conductive and ultrastretchable conductors. *NPJ Flexible Electronics* **5**, 25 (2021).
6. Li, G., Wu, X. & Lee, D.-W. Selectively plated stretchable liquid metal wires for transparent electronics, *Sensors and actuators B: Chemical* **221**, 1114-1119 (2015).
7. Ma, J.-L., Dong, H.-X. & He, Z.-Z. Electrochemically enabled manipulation of gallium-based liquid metals within porous copper. *Materials Horizons* **5**, 675-682 (2018).
8. Kramer, R. K., Boley, J. W., Stone, H. A., Weaver, J. C. & Wood, R. J. Effect of Microtextured surface topography on the wetting behavior of Eutectic gallium-indium alloys. *Langmuir* **30**, 533-539 (2014).
9. Lin, W., Qiu, W., Tuersun, Y., Huang, X. & Chu, S. Ultrastrong spontaneous surface wetting of room temperature liquid metal on treated metal surface. *Advanced Materials interfaces* **8**, 2100819 (2021).
