# Supplementary material for: Imbibition-induced selective wetting of liquid metal
Source: Nat Commun. 2022 Aug 13;13:4763. doi: 10.1038/s41467-022-32259-3 (PMC9376080; doi:10.1038/s41467-022-32259-3)
Supplement: Supplementary file 3 — Description of Additional Supplementary Files [file 41467_2022_32259_MOESM3_ESM.pdf]

Description of Additional Supplementary Files

File Name: Supplementary Movie 1
Description: A top-view video showing imbibition-induced EGaIn wetting

File Name: Supplementary Movie 2
Description: A video showing propagation of imbibition front of EGaIn.
